# HAX1 in cancer: from molecular scaffolding to therapeutic targeting

**DOI:** 10.3389/fonc.2026.2000988

**Published:** 2026-10-21

**Authors:** Tonglin Sun, Dan Xu, Tianci Zhou, Feng Luo, Yan Zhang

**Affiliations:** 1 Department of Medical Oncology, Cancer Center, West China Hospital, Sichuan University, Chengdu, Sichuan, China; 2 Lung Cancer Center/Lung Cancer Institute, West China Hospital, Sichuan University, Chengdu, Sichuan, China; 3 Department of Oncology, The Affiliated Hospital of Southwest Medical University, Luzhou, Sichuan, China

**Keywords:** HAX1, molecular scaffolding, prognostic biomarkers, therapeutic target, treatment resistance, tumor progression, ubiquitination

## Abstract

HCLS1-associated protein X-1 (HAX1) is widely expressed in human tissues and was first identified as a binding partner of hematopoietic cell-specific protein 1. Through interactions with proteins and RNAs, HAX1 regulates gene expression and contributes to cell survival and cytoskeletal remodeling. Recent studies have reported increased HAX1 expression in several malignant tumors, including breast, gastric, colorectal, and other cancers, where it promotes cell survival, proliferation, migration, and invasion. It also supports angiogenesis, hypoxic adaptation, and resistance to anticancer treatment. Noncoding RNAs and ubiquitination regulate HAX1 abundance and molecular state, influencing its cellular functions. Clinical evidence links HAX1 expression and subcellular localization to aggressive clinicopathological features and poor patient outcomes. This supports HAX1 as both a prognostic biomarker and a therapeutic target. This review summarizes the molecular characteristics and regulation of HAX1, and examines how its interactions contribute to tumor progression. It also discusses the implications for cancer prognosis and treatment. Future research should focus on clinical validation and further investigation of HAX1 interactions to guide selective therapeutic targeting.

## 1 Introduction

HCLS1-associated protein X-1 (HAX1) was first identified as an intracellular binding partner of hematopoietic cell-specific protein 1 (HS1), from which its name was derived (1). Encoded by a gene on human chromosome 1q21.3, the canonical HAX1 protein isoform contains 279 amino acids (2, 3). HAX1 is mainly found in mitochondria, but it is also detected in the endoplasmic reticulum (ER), nuclear membrane, and cytoplasm (1). Within the cytoplasm, HAX1 localizes to vesicles and RNA granules, including processing bodies (P-bodies), while in migrating cells it also accumulates near the plasma membrane at the leading edge of lamellipodia (4–6). HAX1 is widely but unevenly expressed across human tissues, with relatively higher RNA levels in skeletal muscle and lower levels in the lung, as shown in **Figure 1** (7). As a multifunctional protein, HAX1 regulates multiple aspects of gene expression, including transcriptional regulation, translation, mRNA binding and processing, and ribosome biogenesis (6, 8, 9). Through these activities, HAX1 profoundly influences cellular functions. Moreover, HAX1 has been implicated in inflammatory disorders, host-virus interactions and hematopoietic/immune diseases, highlighting its broader role in maintaining tissue homeostasis and adapting to external insults (10–13).

**Figure 1:**
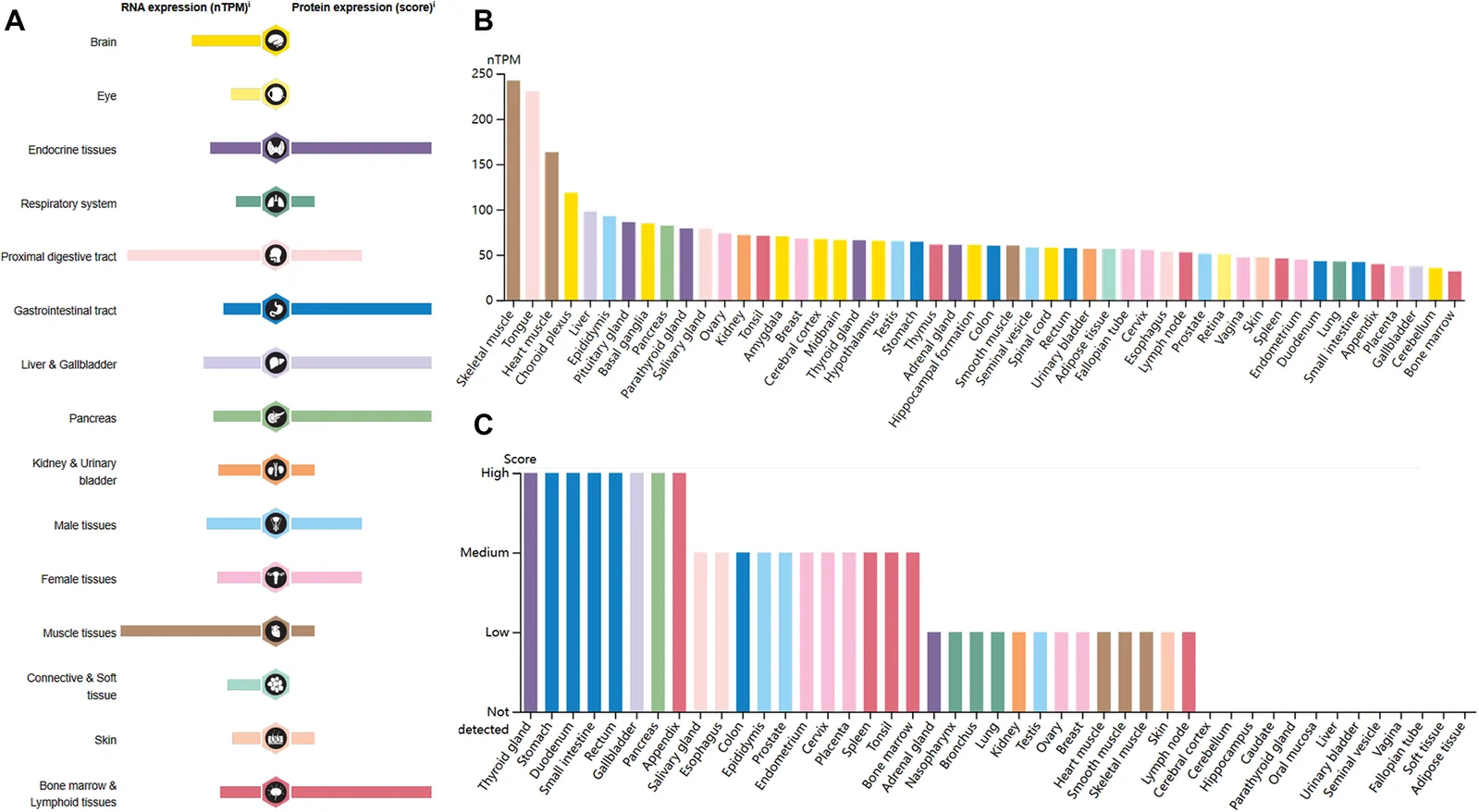
Tissue distribution of HAX1 RNA and protein expression. **(A)** Overview of HAX1 RNA and protein levels across major human tissue groups; **(B)** consensus RNA expression across individual tissues, reported as normalized transcripts per million (nTPM); **(C)** protein expression across individual tissues, categorized as high, medium, low, or not detected. Data were obtained from the Human Protein Atlas.

Malignant tumors are a major challenge to global health, and their morbidity and mortality rates remain high. Traditional therapies such as chemotherapy and radiotherapy are limited by resistance and severe side effects, often resulting in poor prognosis and impaired quality of life. Therefore, an understanding of the molecular mechanisms of tumors and search for new therapeutic targets are vital to improving treatment efficiency and patient survival. Although HAX1 was initially investigated in the hematopoietic system, its expression is not restricted to hematopoietic cells. Subsequent studies reported abnormal HAX1 expression in several malignant tumors, including pharyngeal (14), breast (15), lung (16), esophageal (17), gastric (18), colorectal (19), and liver (20) cancers, which has become a hot research topic within the discipline of tumor biology. This overexpression suggests a role in cancer progression. Functional studies support this view, showing that HAX1 can promote cancer cell growth, metastasis, and angiogenesis by modulating key signaling pathways (21). At the molecular level, HAX1 exerts its functions through interactions with diverse proteins, yet how it contributes to carcinogenesis and malignant progression remains incompletely defined.

These findings provide a basis for reviewing the molecular roles and translational value of HAX1 in cancer. This review therefore summarizes the molecular characteristics, dysregulation, and oncogenic functions of HAX1 and evaluates its potential as a cancer biomarker and therapeutic target. To integrate these findings, we build on the established role of HAX1 as a scaffold that binds various protein partners. Its functions depend on the cellular context. In this context, expression level regulation and ubiquitination alter HAX1 abundance and molecular state in tumor cells. Moreover, its subcellular localization and interactions with distinct binding partners shape its roles in cell survival, proliferation, migration, hypoxic adaptation, and treatment resistance.

## 2 Basic structure and function of HAX1

HAX1 lacks a defined catalytic domain and is predominantly disordered in its unbound state. Upon binding to lipid membranes, however, it can acquire local secondary structures (3). Alternative splicing also generates several HAX1 isoforms, with variant I predominating in human tissues (2, 22). These isoforms differ in sequence and subcellular distribution, which may contribute to differences in their functions (4). Structurally, HAX1 contains an acidic region, a PEST sequence, and two functional nuclear export signals. The PEST sequence promotes polyubiquitination and proteasomal degradation, while the export signals support exportin 1 (XPO1)-dependent shuttling between the nucleus and cytoplasm (23, 24). Consistent with this shuttling, HAX1 shows differences in nuclear and cytoplasmic distribution in tumor tissues (22). Beyond this subcellular distribution, it also functions as an intrinsically disordered scaffold, binding multiple protein partners through distinct regions of its sequence and facilitating protein complex assembly. These regions mediate homo- and heterodimerization and facilitate the assembly of diverse protein complexes. For example, HAX1 binds to the β6 cytoplasmic tail and regulates clathrin-mediated αvβ6 endocytosis, thus promoting tumor progression (25). Furthermore, HAX1 interacts with HS1 and the F-actin-binding protein cortactin, linking it to cytoskeletal remodeling (1, 26). Its interactions with the sarcoplasmic/endoplasmic reticulum Ca²^+^-ATPase 2 (SERCA2) and the mitochondrial chaperone CLPB further connect HAX1 to calcium homeostasis and mitochondrial proteostasis, respectively (27, 28). Despite lacking a canonical RNA-binding domain, HAX1 binds to the 3′-untranslated regions (3′-UTRs) of vimentin and DNA polymerase β mRNAs and associates with a broad range of RNAs involved in ribosomal RNA processing, ribosome assembly, cytoskeleton organization, and protein translation (8, 9, 29, 30).

Beyond cancer, HAX1 contributes to calcium homeostasis in cardiomyocytes, neuronal survival, and mitochondrial proteostasis during neutrophil differentiation. Its protective effects may partly depend on its interactions with chaperones. HAX1 has been proposed to help these proteins bind their targets, linking its molecular interactions to protein homeostasis and stress protection (31). These roles help explain how HAX1 can support cancer cell survival under stress and provide a biological basis for understanding its oncogenic functions.

## 3 Dysregulation of HAX1 in cancer

### 3.1 RNA-mediated regulation

The cellular functions of HAX1 depend partly on its intracellular abundance, which is regulated post-transcriptionally by noncoding RNAs in cancer. HAX1-targeting microRNAs (miRNAs) generally bind to the 3′-UTR of HAX1 mRNA and suppress its expression, while long noncoding RNAs (lncRNAs) and circular RNAs (circRNAs) can sequester these miRNAs and relieve this repression. In hepatocellular carcinoma (HCC), Zha et al. (32) demonstrated that miR-125a-5p directly targets HAX1, suppressing tumor cell growth, migration, and epithelial-mesenchymal transition. Wu et al. (33) subsequently identified the lncRNA cytoskeleton regulator RNA (CYTOR) as a regulator of the same axis. By attenuating miR-125a-5p-mediated repression, CYTOR preserved HAX1 expression and reduced mitochondrial caspase-9 activation and apoptosis. A similar mechanism operates in esophageal squamous cell carcinoma (ESCC), where circGFPT1 sequesters miR-142-5p and increases HAX1 expression (34). Conversely, miR-125b directly targets HAX1 and limits cell proliferation and invasion (35). Restoring miR-125b-mediated repression of HAX1 in breast cancer cells also increases their sensitivity to doxorubicin (36).

HAX1 can also sustain its own expression through reciprocal RNA regulation. In nasopharyngeal carcinoma (NPC), HAX1 binds insulin-like growth factor 2 mRNA-binding protein 1 (IGF2BP1) mRNA and enhances its stability and translation. IGF2BP1, an N6-methyladenosine (m6A) reader, in turn stabilizes HAX1 mRNA through an m6A-dependent mechanism (37). This positive feedback loop increases HAX1 abundance and links RNA regulation to multiple downstream cellular processes.

### 3.2 Ubiquitin-mediated regulation

RNA-mediated mechanisms regulate HAX1 production, while the ubiquitin system controls its stability and turnover. Specifically, the PEST sequence of HAX1 promotes lysine 48 (K48)-linked polyubiquitination, committing the protein to proteasomal degradation (23). In mantle cell lymphoma, protein kinase C delta (PRKCD) phosphorylates both HAX1 and the E3 ubiquitin ligase substrate receptor F-box protein 25 (FBXO25), promoting FBXO25-dependent HAX1 degradation. Disruption of this axis causes HAX1 accumulation and attenuates the apoptotic response of lymphoma cells (38). An opposing mechanism stabilizes HAX1 in colorectal cancer (CRC). Eukaryotic translation initiation factor 3 subunit H (EIF3H) acts as a deubiquitinase that removes β-transducin repeat-containing protein (β-TrCP)-mediated polyubiquitin chains from HAX1. Stabilized HAX1 then promotes formation of the RAF1–MEK1–ERK1 complex (39). Ryu et al. (40) further showed that ribosomal protein S4 X-linked (RPS4X) disrupts Cullin1–Skp1 binding and suppresses Skp1–Cullin1–F-box (SCF) E3 ubiquitin ligase complex-mediated HAX1 degradation. RPS4X expression also increased resistance to doxorubicin-induced apoptosis in HeLa cells, although both HAX1 and myeloid cell leukemia 1 (MCL1) were stabilized. These studies place HAX1 stability under the joint control of degron-dependent ubiquitination and deubiquitination. Perturbation of these mechanisms can stabilize HAX1 and alter cellular responses to apoptotic stress.

HAX1 exchanges dynamically between the cytoplasm and P-bodies, and its recruitment to these RNA granules depends on mRNA availability (4). Ubiquitination provides another means of regulating this behavior. Unlike degradation-promoting K48-linked chains, lysine 63 (K63)-linked ubiquitin chains drive HAX1 condensation and stress adaptation. Under energy stress, the E3 ubiquitin ligase tripartite motif-containing 23 (TRIM23) catalyzes K63-linked polyubiquitination of HAX1, inducing liquid-liquid phase separation and assembly of P-bodies. Once formed, P-bodies suppress global protein synthesis and support CRC cell proliferation during nutrient deprivation. In patient samples, increased cytoplasmic TRIM23 localization, HAX1 expression, and HAX1 ubiquitination are also associated with poor outcomes (41). Protein degradation, stabilization, and condensation therefore represent distinct ways to regulate HAX1 abundance and molecular state.

## 4 HAX1 promotes tumor progression

### 4.1 Cell survival and proliferation

Regulation by RNA and ubiquitin influences HAX1 abundance and molecular state, with consequences for tumor cell survival and proliferation. HAX1 protects cells from apoptosis through multiple mitochondrial and ER pathways. During stress, its interaction with the non-receptor tyrosine kinase c-Abl supports reactive oxygen species (ROS) clearance (42). HAX1 also binds apoptosis antagonizing transcription factor (AATF/Che-1), a stress-response protein that regulates p53 activity and mTOR signaling (43, 44). Oxidative stress increases this interaction in MCF7 breast cancer cells, further supporting a role for HAX1 in stress adaptation and cell survival (45). Additionally, HAX1 regulates SERCA2 levels and calcium stores, and suppresses caspase-9 activation (27, 46). These effects limit cell death caused by oxidative stress, disturbed calcium homeostasis, and mitochondrial apoptosis. Consistent with this protective role, HAX1 knockdown increased mitochondrial apoptosis in uveal melanoma cells (47). In cervical carcinoma cells, HAX1 likewise supports cell survival and protects against human papillomavirus 16 (HPV16) E2-induced mitochondrial apoptosis (48). In addition to these mitochondrial and ER effects, HAX1 supports cell survival by modulating signaling pathways that regulate apoptosis. In non-small cell lung cancer (NSCLC), HAX1 sustains AKT/mTOR pro-survival signaling and MDM2-mediated suppression of p53. Consequently, HAX1 knockdown restores p53 activity and induces apoptosis (16). A similar role in promoting cell survival through AKT1 signaling has been reported in glioblastoma (49).

Beyond suppressing apoptosis, stabilized HAX1 can also reinforce proliferative signaling. EIF3H-stabilized HAX1 in CRC directly promotes RAF1 dimerization and assembly of the RAF1–MEK1–ERK1 complex, leading to ERK1/2 activation and increased tumor cell proliferation (39). A role for HAX1 in promoting proliferation has also been reported in cervical carcinoma cells (50). Gene silencing experiments support the same conclusion. Knockdown assays in gastric cancer, cutaneous squamous cell carcinoma (cSCC), and hypopharyngeal squamous cell carcinoma further show that HAX1 depletion induces cell cycle arrest, reduces proliferation, and increases apoptosis (14, 18, 51).

### 4.2 Migration and invasion

Beyond supporting survival and proliferation, tumor cells can further exploit HAX1 to remodel cell adhesion and actin networks, thereby enhancing cell motility. HAX1 binds the β6 cytoplasmic tail of the epithelial adhesion receptor integrin αvβ6 and promotes its clathrin-mediated endocytosis. HAX1 knockdown or blockade of this interaction suppresses the migration and invasion of αvβ6-positive cancer cells (25). In MCF7 breast cancer cells, HAX1 binds the scaffold protein IQ motif-containing GTPase-activating protein 1 (IQGAP1) and recruits it to the leading edge and focal adhesions, sustaining focal adhesion turnover and directional migration (5). In ovarian carcinoma cells, HAX1 scaffolds the Rac1–cortactin complex required for lysophosphatidic acid-induced migration (26). A further interaction connects HAX1 to nucleotide-binding oligomerization domain-containing protein 1 (NOD1). In cervical carcinoma cells, HAX1 and NOD1 cooperate to promote migration independently of receptor-interacting serine/threonine-protein kinase 2 (RIPK2) and nuclear factor kappa B (NF-κB) signaling (52). By contrast, HAX1 promotes migration and invasion in HCC through NF-κB-dependent epithelial-mesenchymal transition (20). HAX1 enhances cell motility through distinct mechanisms in different tumor contexts.

### 4.3 Hypoxic adaptation and angiogenesis

HAX1 contributes to hypoxic adaptation and angiogenesis through distinct mechanisms. In glioma cells, hypoxia-inducible factor 1 alpha (HIF-1α) increases HAX1 expression. HAX1 then promotes AKT-dependent phosphorylation of dynamin-related protein 1 (Drp1) and mitochondrial fission, allowing glioma cells to maintain growth and invasive behavior under hypoxia (53). At the vascular level, tumor-derived extracellular vesicles transfer HAX1 to endothelial cells, where it increases the translation of integrin subunit beta 6 (ITGB6) and activates focal adhesion kinase (FAK). The resulting increase in endothelial migration and tube formation promotes angiogenesis, while HAX1-rich vesicles also increase metastasis *in vivo* (54). Moreover, HAX1 has been linked to AKT-dependent angiogenesis in HCC models (21).

### 4.4 Therapeutic resistance

The stress protection functions of HAX1 also reduce tumor cell sensitivity to cancer treatment. In cisplatin-resistant bladder cancer cells, miR-325 is downregulated. Restoring miR-325 suppresses HAX1, disrupts the mitochondrial membrane potential, activates caspase-9, caspase-7, and caspase-3, and resensitizes these cells to cisplatin-induced apoptosis (55). A similar mitochondrial mechanism operates in doxorubicin-resistant breast cancer cells. In these cells, miR-125b-mediated repression of HAX1 increases ROS accumulation and mitochondrial damage, restoring doxorubicin sensitivity (36). These findings connect HAX1-dependent mitochondrial protection to chemoresistance.

HAX1 promotes resistance through additional cell death pathways. In NPC, reciprocal stabilization of HAX1 and IGF2BP1 mRNAs increases HAX1 abundance, disrupting Rab7a-dependent autophagic trafficking. HAX1 then recruits the E3 ubiquitin ligase NEDD4 to accelerate Rab7a degradation and competes with the soluble N-ethylmaleimide-sensitive factor attachment protein receptor (SNARE) complex for Rab7a binding. These effects block autophagosome-lysosome fusion and increase cisplatin resistance (37). This protection extends to ferroptosis in HCC. HAX1 maintains iron homeostasis and the glutathione (GSH) and glutathione peroxidase 4 (GPX4) antioxidant system. Its depletion increases ferrous iron and lipid peroxidation and sensitizes tumor cells to imidazole ketone erastin (IKE) (56).

## 5 HAX1 as a biomarker in cancer

Across studies, HAX1 is more abundant in tumor than in adjacent or normal tissue, with clear contrasts reported in NPC and cSCC. HAX1 positivity was detected in 74.4% of NPC versus 20.9% of non-tumor nasopharyngeal tissues (57). In cSCC, moderate or strong staining was present in 61.7% of tumors and 3.3% of normal skin samples (51). These differences support further evaluation of HAX1 as a diagnostic marker by measuring its level in different malignant tumors. Meanwhile, HAX1 is enriched in serum extracellular vesicles from patients with NPC compared with healthy donors, making circulating vesicular HAX1 a candidate for liquid biopsy studies (54, 57).

HAX1 also has potential as a prognostic biomarker. High HAX1 expression was reported as an independent adverse prognostic factor in gastric cancer (18), CRC (19), NPC (57), laryngeal carcinoma (58), and glioma (59). The survival difference was especially clear in gastric cancer, where patients with high HAX1 expression had a significantly lower 3-year overall survival (OS) rate than those with low expression (41.5% vs. 64.6%, P = 0.0001) (18). High expression was also associated with poor survival in multiple myeloma (60). Beyond survival, clinical studies have linked HAX1 expression to features of disease progression. In laryngeal carcinoma, HAX1 expression correlated with T classification, lymph node metastasis, and clinical stage (58). In HCC, it correlated with tumor size and histological grade (61). Assessing HAX1 alongside other molecular markers may provide further prognostic information. In ESCC, HAX1 expression alone was associated with tumor size, pT stage, lymph node metastasis, and TNM stage. When integrin β6 expression was considered alongside HAX1, concurrent positivity identified patients with the poorest survival (62). Combined expression was also linked to prognosis at the RNA level. Colorectal tumors with metastasis had higher transcript levels of both HAX1 and hyaluronan-mediated motility receptor (HMMR). The two genes showed a synergistic association with metastasis-free survival (MFS) (63).

The prognostic value of HAX1 also depends on its location within cells. In breast cancer, cytoplasmic HAX1 independently predicted distant recurrence, whereas nuclear HAX1 did not (15). This distinction suggests that measuring total HAX1 expression alone cannot provide enough information for recurrence risk. Genetic variation in HAX1 may provide additional prognostic information. In resected early-stage NSCLC, the rs11265425T>G variant increased HAX1 promoter activity and was associated with shorter disease-free survival, mainly in adenocarcinoma (64). The biological roles and clinical significance of HAX1 across different cancers are summarized in **Table 1**.

**Table 1:** Biological and clinical significance of HAX1 dysregulation in human cancers.

Tumor type	Expression status	Molecular context and core mechanism	Clinicopathological features	Prognostic value	Intervention and response	References
Bladder cancer	Increased in cisplatin-resistant cells	Loss of miR-325 increases HAX1 and preserves mitochondrial integrity	–	–	miR-325 restoration or HAX1 suppression restores cisplatin sensitivity	(55)
Breast cancer	Upregulated	IQGAP1-dependent focal adhesion turnover; miR-125b/HAX1 control of mitochondrial ROS	Distant metastatic recurrence	Independently predicts recurrence (cytoplasmic, but not nuclear)	HAX1 repression restores doxorubicin sensitivity; TAT-RGCT-GFP blocks migration	(5, 15, 22, 36)
Cervical squamous carcinoma	Upregulated	NOD1-dependent migration without RIPK2 or NF-κB; inhibition of mitochondrial apoptosis	–	–	–	(48, 50, 52)
Colorectal cancer (CRC)	Upregulated	EIF3H stabilizes HAX1; TRIM23-mediated K63 ubiquitination drives P-body condensation; HMMR coexpression	Nodal metastasis; advanced TNM stage; metastatic disease	Independent poor OS; combined HAX1/HMMR is linked to MFS	HAX1 depletion restores camptothecin-induced apoptosis	(19, 39, 41, 63, 66)
Cutaneous squamous cell carcinoma (cSCC)	Upregulated	Promotes proliferation and motility and suppresses apoptosis	Greater thickness; poor differentiation; advanced TNM stage	–	–	(51)
Esophageal squamous cell carcinoma (ESCC)	Upregulated	HAX1/integrin β6 coexpression; circGFPT1/miR-142-5p and miR-125b regulate HAX1	Tumor size; pT stage; nodal metastasis; TNM stage	Poor OS; dual HAX1/integrin β6 positivity identifies the poorest group	–	(17, 34, 35, 62)
Gastric cancer	Upregulated	Promotes cell cycle progression and suppresses apoptosis	Poor differentiation; vascular tumor thrombi; nodal metastasis; advanced TNM stage	Independent poor survival	–	(18)
Glioma	Upregulated	AKT1-dependent survival; HIF-1α/AKT/Drp1-mediated mitochondrial fission under hypoxia	Poor differentiation; higher WHO grade	Independent poor survival	–	(49, 53, 59)
Hepatocellular carcinoma (HCC)	Upregulated	CYTOR/miR-125a-5p control; AKT and NF-κB signaling; GSH/GPX4-dependent ferroptosis defense	Tumor size; histological grade; cirrhosis	Independent poor survival	HAX1 silencing increases sensitivity to imidazole ketone erastin	(20, 21, 32, 33, 56, 61)
Laryngeal carcinoma	Upregulated	–	T classification; nodal metastasis; clinical stage; pathology	Poor OS	–	(58)
Nasopharyngeal carcinoma	Upregulated	IGF2BP1/HAX1 RNA feedback blocks Rab7a-dependent autophagic flux; vesicular HAX1 activates ITGB6/FAK	Nodal and distant metastasis; advanced cTNM stage	Independent poor prognosis	HAX1 knockdown enhances cisplatin response in cells and xenografts	(37, 54, 57)
Non-small cell lung cancer (NSCLC)	Upregulated	AKT/mTOR activation and MDM2-mediated p53 suppression; rs11265425 G increases promoter activity	Larger tumor; lymphatic metastasis; advanced TNM stage	Poor survival; rs11265425 G predicts shorter DFS after resection	–	(16, 64)
Oral squamous cell carcinoma	Increases with progression to invasive carcinoma	Regulation of clathrin-mediated endocytosis of αvβ6 integrins	–	–	HAX1 peptide blocks αvβ6-dependent invasion	(25)
Ovarian carcinoma	Upregulated	Scaffolds Rac1/cortactin; RNF217 reduces HAX1 stability	–	–	Competitive HAX D4 fragment reduces LPA-induced migration by more than 75%	(26, 65)
Prostate cancer	Upregulated	Suppresses caspase-9-dependent mitochondrial apoptosis	–	–	–	(46)

DFS, disease-free survival; MFS, metastasis-free survival; IKE, imidazole ketone erastin; LPA, lysophosphatidic acid; OS, overall survival; PDX, patient-derived xenograft; ROS, reactive oxygen species; WHO, World Health Organization.

## 6 HAX1-associated therapeutic vulnerabilities in cancer

In preclinical models of CRC (39), glioma (53), NSCLC (16), NPC (37), HCC (56), and cSCC (51), HAX1 downregulation directly suppresses tumor growth, invasion and therapeutic resistance, supporting its potential as a therapeutic target. However, its lack of a catalytic domain, together with its reliance on protein interactions and scaffolding, renders conventional enzyme-directed inhibition challenging. Therapeutic strategies should therefore focus on disrupting complexes containing HAX1 or blocking its abnormal stabilization and stress-induced condensation.

Disrupting HAX1 interactions provides functional evidence for therapeutic intervention in cancer. In oral squamous cell carcinoma, a cell-penetrating HAX1 peptide competitively displaced HAX1 from integrin β6. This blocked integrin αvβ6 internalization and reduced cell motility and invasion (25). A similar strategy was tested in ovarian carcinoma cells by ectopic expression of the competitive HAX1 fragment HAX D4. The fragment prevented formation of the Rac1 and cortactin complex and reduced lysophosphatidic acid-induced migration by more than 75% (26). Meanwhile, the interaction between HAX1 and IQGAP1 provides another potential target. The HAX1 C terminus binds the Ras GTPase-activating protein-related C-terminal domain of IQGAP1, and perturbing this interaction impairs focal adhesion turnover and directional migration (5). These results identify integrin β6, IQGAP1, Rac1, and cortactin as candidate partners for therapies directed at HAX1.

Targeting the regulators of HAX1 stability and condensation offers a second route. In CRC, EIF3H depletion restores HAX1 ubiquitination and weakens the assembly of the RAF1–MEK1–ERK1 complex. At the pathway level, the Wnt inhibitor LGK974 and the MEK inhibitor trametinib synergistically suppress tumor growth in EIF3H-high patient-derived xenografts, supporting the therapeutic potential of targeting signaling pathways linked to HAX1 stabilization (39). Energy stress regulates HAX1 through a different ubiquitin signal. Blocking TRIM23-mediated K63-linked ubiquitination impairs HAX1 condensation and P-body formation, thereby suppressing tumor growth (41). The RNF217 findings further support targeting HAX1 stability, as RNF217 overexpression reduced HAX1 protein stability and suppressed tumor growth in ovarian cancer (65).

Moreover, reducing HAX1 abundance may improve the effectiveness of other anticancer treatments. HAX1 knockdown in NPC acts synergistically with cisplatin in cultured cells and enhances its antitumor activity in xenograft models (37). In HCC, HAX1 silencing sensitizes tumor cells to ferroptosis inducers (56). Similarly, HAX1 depletion sensitizes SW480 CRC cells to camptothecin-induced apoptosis, reversing the resistant phenotype conferred by HAX1 overexpression (66). These findings support combining HAX1 suppression with treatments that induce tumor cell death. A related strategy could be explored in hypoxic glioma, where HAX1 depletion reduces AKT and Drp1 activity and restricts xenograft growth, providing a rationale for combining HAX1 suppression with inhibition of mitochondrial fission (53). However, this combination remains to be tested directly. Beyond evaluating therapeutic efficacy, the development of these strategies must also account for the normal functions of HAX1. Germline loss-of-function variants in HAX1 cause severe congenital neutropenia, raising safety concerns for systemic HAX1 inhibition (67). Therapeutic development must therefore distinguish tumor dependencies from HAX1 functions required by normal hematopoietic cells.

## 7 Conclusion and perspectives

This review integrates evidence that HAX1 acts as a scaffold whose functions depend on cellular context. Its disordered structure enables it to bind diverse proteins and RNAs, while noncoding RNA and ubiquitin pathways regulate its abundance and molecular state. These regulatory layers, together with its interactions with different partners, influence the cellular functions of HAX1. Through its scaffolding role, HAX1 facilitates the assembly of distinct protein complexes that contribute to tumor growth, dissemination, and treatment resistance. Moreover, HAX1 expression is elevated in diverse cancers compared with adjacent normal tissues. HAX1 overexpression often accompanies aggressive clinicopathological features and poor outcomes, supporting its evaluation as both a diagnostic marker and a prognostic biomarker. Beyond its biomarker value, HAX1 offers several points for therapeutic intervention that vary among tumors. Identifying the relevant subcellular localization, modification state, and interaction network could enable selective targeting while preserving HAX1 function in normal hematopoietic cells.

Although considerable progress has been made in understanding the role of HAX1 in cancer, further studies are needed to establish its clinical value. Existing clinical evidence relies largely on retrospective analyses, often focusing on HAX1 expression, while the pathways underlying its effects in individual tumor types remain incompletely understood. Prospective studies should therefore determine whether HAX1 adds prognostic information beyond established clinicopathological factors and whether changes in its expression reflect treatment response or recurrence. In this regard, assessing its subcellular localization and molecular partners alongside expression may help explain differences in its clinical significance across tumors. A clearer understanding of these mechanisms would also guide therapeutic development by identifying which HAX1 interactions or regulatory changes are required for tumor progression. Disrupting these interactions or preventing abnormal HAX1 stabilization could offer opportunities for selective intervention. In addition, testing these approaches in tumor models alongside normal hematopoietic cells would help establish whether their antitumor effects can be achieved while preserving essential HAX1 functions.

The interaction network of HAX1 also raises a more specific question. HAX1 supports Rac1 and cortactin complex formation during migration, while recent work on HS1BP3 links its interaction with cortactin to TKS5 localization, cell secretion, and matrix degradation (26, 68). Although HAX1, also known as HS1BP1, and HS1BP3 were both identified as HS1 binding partners, their functional relationship remains unclear. Their shared interaction with cortactin, however, suggests a possible connection and prompts the question of whether they bind overlapping regions and compete for access to this protein. Comparing their binding sites and testing whether either protein alters the binding of the other could clarify how these interactions influence tumor progression.
